# Establishment of the Alabama Hereditary Cancer Cohort ‐ strategies for the inclusion of underrepresented populations in cancer genetics research

**DOI:** 10.1002/mgg3.443

**Published:** 2018-07-01

**Authors:** Madison R. Bishop, Amit Shah, Melissa Shively, Anna L. W. Huskey, Sophonie M. Omeler, Erin P. Bilgili, Ebony Jackson, Kathleen Daniell, Elizabeth Stallworth, Stephanie Spina, Kasey Shepp, Sydney Bergstresser, Amber Davis, Holly Dean, Jantunn Gibson, Brandon Johnson, Nancy D. Merner

**Affiliations:** ^1^ Department of Drug Discovery and Development Harrison School of Pharmacy Auburn University Auburn Alabama; ^2^ Department of Pathobiology College of Veterinary Medicine Auburn University Auburn Alabama; ^3^ East Alabama Medical Center, Cancer Center Opelika Alabama; ^4^ Department of Human Development and Family Studies College of Human Sciences Auburn University Auburn Alabama

**Keywords:** African American, biobank, hereditary breast cancer, recruitment, underrepresented individuals

## Abstract

**Background:**

Historically, groups that are most susceptible to health and healthcare disparities have been underrepresented in medical research. It is imperative to explore approaches that can facilitate the recruitment of underrepresented individuals into research studies.

**Methods:**

Two approaches, hospital and community‐based recruitment (CBR), were developed and implemented over 36 months to study the genetics of hereditary breast cancer and associated cancers in Alabama, a medically underserved state with double the national percentage of self‐identifying African Americans, establishing the Alabama Hereditary Cancer Cohort.

**Results:**

Overall, 242 individuals enrolled. This included 84 cancer probands through hospital recruitment, as well as 76 probands and 82 family members through CBR. Eighty‐one percent of the study participants’ counties of residence are completely medically underserved. Furthermore, African Americans represent 26% of the hospital probands compared to 49% and 70% of the probands and family members who, respectively, enrolled through CBR.

**Conclusion:**

Although both recruitment mechanisms were instrumental, the unique trust building, educational, and traveling components of CBR facilitated the enrollment of African Americans resulting in large families for genetic analyses. The ultimate goal is to gain insight from these rudimentary efforts in order to expand recruitment and accrue a unique resource for cancer genetics research.

## 1 INTRODUCTION

Health and healthcare disparities have been an enduring and tenacious issue in the United States. Many groups are vulnerable to such disparities, including (but not limited to) ethnic minorities and individuals of lower socioeconomic status, as well as people in particular geographic locations. Since such groups are not always mutually exclusive, many subgroups of vulnerable populations exist (Frist, 2005; Ubri & Artiga, 2016; Wong, 2015). Historically, groups that are most susceptible to health and healthcare disparities have been underrepresented in medical research (Brown, 2004; Farmer, Jackson, Camacho, & Hall, 2007; Konkel, 2015). Even today, in the era of genomic sequencing and precision medicine/health, this fact still holds true (Ashley, 2016; Bustamante, Burchard, & De la Vega, 2011; Spratt et al., 2016). This is extremely unfortunate since ethnic diversity, socioeconomic status, and geography all play a role in disease susceptibility, progression, and outcomes (Konkel, 2015; Spratt et al., 2016; Tan, Mok, & Rebbeck, 2016; Wong, 2015). If underrepresented individuals continue to be overlooked as research participants, progress in precision medicine/health will be limited, and health disparities will be exacerbated (Bustamante et al., 2011; Konkel, 2015; Spratt et al., 2016; Streicher et al., 2011).

Involving underrepresented individuals in research studies is not a simple task; in fact, the time and effort that must be invested for success can be greatly underappreciated (Brown, 2004; Farmer et al., 2007; Taylor, 2009). It requires overcoming barriers and addressing informational, logistical, sociocultural, and attitudinal factors that could otherwise negatively influence research participation (Brown, 2004; Farmer et al., 2007). There are examples of the successful recruitment of individuals in rural areas (Newman et al., 1995) and collaborative efforts for the study of minority groups (Palmer, Ambrosone, & Olshan, 2014), but these efforts are generally the exceptions. It is imperative that researchers continue to explore methods that will help facilitate the recruitment of underrepresented individuals since the implementation of well‐executed and appropriate recruitment efforts is key to true inclusion (Farmer et al., 2007).

**Figure 1:**
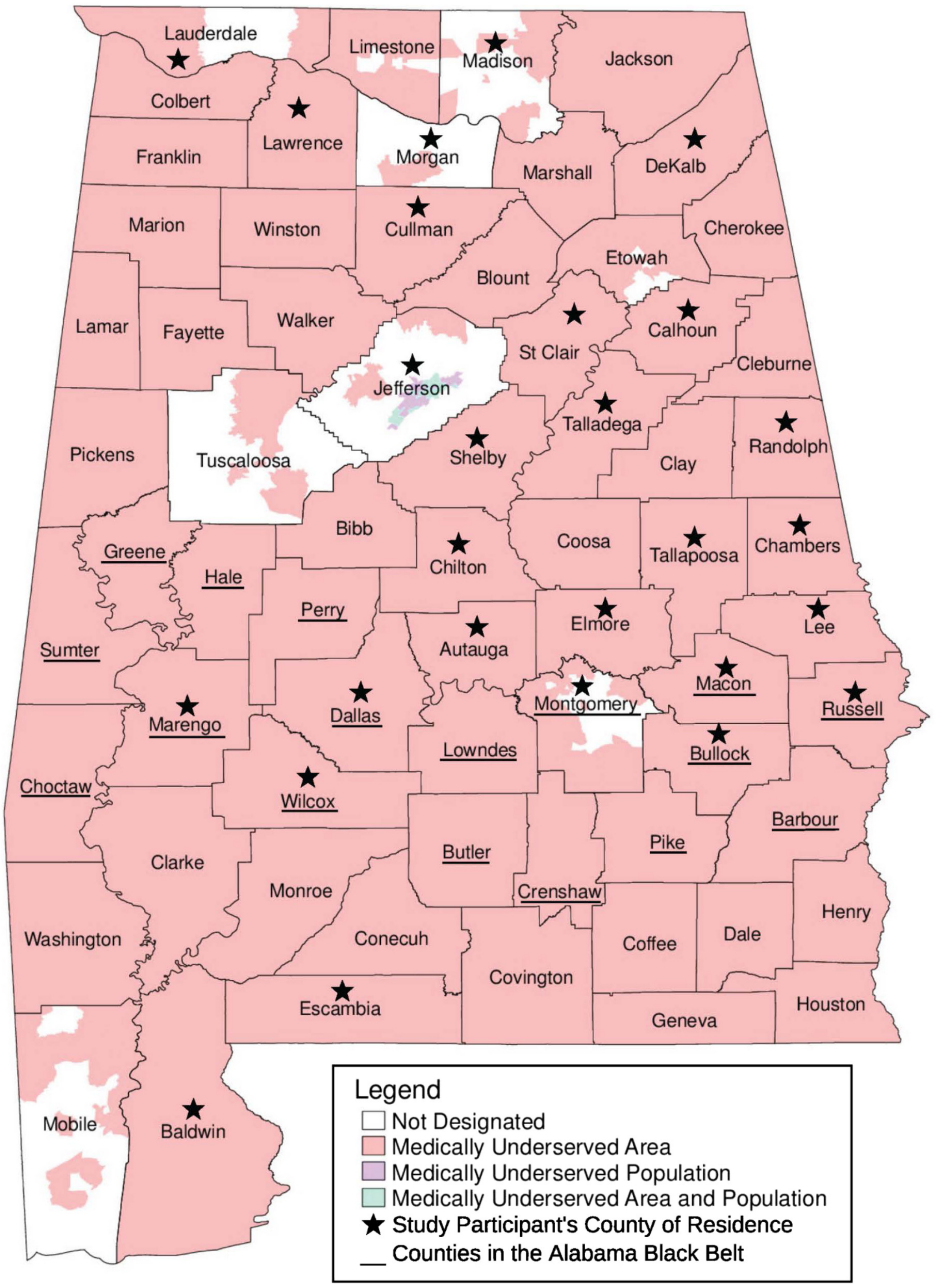
Map of Alabama divided into counties. Medically underserved areas and populations, and counties of residence of current study participants are highlighted; see figure legend. The original map was obtained from the Alabama Department of Public Health (ADPH) website (http://www.alabamapublichealth.gov/ruralhealth/assets/MUAPMap.pdf) with permission to use to demonstrate recruitment progress. According to the ADPH, medically underserved areas are “a measure of the number of health professionals and certain health outcomes that demonstrate a lack of access and impact on the health of the community. Medically underserved populations are very similar to medically underserved areas except that they are designating the low‐income population rather than the geographical region” (http://www.alabamapublichealth.gov/ruralhealth/assets/MUAP_101.pdf)

Herein, we describe the recruitment approaches and biobanking for the Alabama Hereditary Cancer Cohort to facilitate the genetic analyses of hereditary breast cancer (BC) and associated cancers such as ovarian (OvC) and prostate cancer (PC) (Chandler, Bilgili, & Merner, 2016) in Alabama. Over 60% of the Alabama population is medically underserved; this includes the entire population of 85% of its counties, most of which are rural (Figure 1) (Alabama Department of Public Health, Rural Health, Shortage Area Designations, 2017; Susan G. Komen North Central Alabama Affiliate 2015 Community Profile, 2015). Furthermore, the percentage of the Alabama population who self‐describe as being black or African American is nearly double that of the national population (26.8% vs. 13.6%, respectively) with a predominantly African American population residing within the Alabama Black Belt region, an area associated with low economic status that encompasses 25% of the state's counties (Figure 1) (Gyawu, Quansah, Fall, Gichuhi, & Bovell‐Benjamin, 2015; Rastogi, Johnson, Hoeffel, & Drewery, 2010). Thus, our recruitment mechanisms, which include standard hospital recruitment along with strategic and adaptive community‐based recruitment (CBR), target underprivileged and minority groups in Alabama and aim to create a unique cohort of underrepresented individuals to study cancer genetics and disparities.

## 2 METHODS

### 2.1 Ethical compliance

Two different Auburn University (AU; Auburn, Alabama; Lee County; Figure 1) Institutional Review Board (IRB) approved protocols, hospital recruitment (AU IRB #14‐232; approved January 2015) and CBR (AU IRB #15‐111; approved March 2015), were designed to recruit and enroll BC, OvC, and PC affected individuals/families for the establishment of the Alabama Hereditary Cancer Cohort.

Study criteria include individuals: (1) diagnosed with BC, OvC, or PC (at any age) who have a family history of cancer, or (2) diagnosed with BC, OvC, or PC under the age of 45 years without a family history of cancer. Furthermore, both cancer‐affected and unaffected family members of each study participant can join the study. The first cancer‐affected individual to enroll in the study from each family is defined as the family proband. Recruitment and enrollment efforts were carried out over a 36‐month period from January 2015 to December 2017.

### 2.2 Hospital recruitment

A general AU hospital recruitment protocol was initiated based on a partnership with the Cancer Center of East Alabama Medical Center (EAMC) and the approval of EAMC IRB protocol, 14‐03‐E. EAMC is located in Opelika, Alabama (Lee County) and serves six Alabama counties (Lee, Chambers, Tallapoosa, Macon, Russell, and Randolph counties; Figure 1). Overall, the hospital recruitment protocol was designed to allow the recruitment and enrollment of patients who fit the study criteria at current and future collaborating hospitals. The recruitment effort involves a designated hospital staff member, typically, a project‐assigned research nurse, screening patients for eligibility. At EAMC, a part‐time research nurse carried out this effort by screening the medical records of individuals on the cancer center's weekly schedule. Upon identification of potential study participants, the research nurse would contact those individuals to inform them of the study and schedule an enrollment appointment at the hospital, if interested. Upon consent, hospital medical records are accessed for information pertaining to the cancer diagnoses; furthermore, demographic information is recorded along with the participant's personal and family history of cancer and other cancer risk factors (i.e., number of children, breastfeeding habits, etc.). A pedigree is drawn to detail this information. Additionally, a blood sample is provided for DNA extraction and subsequent genetic analysis (AU IRB #14‐335). The collected information is subject to the confidentiality and privacy regulations of the recruiting hospital. The hospital removes each participant's name and assigns a specific alpha‐numeric code to the collected blood sample and corresponding paper work/information that is transferred to AU. Lastly, each study participant agrees to future contact for additional sampling and/or information regarding cancer risk and updates.

### 2.3 CBR

The CBR protocol was established to engage individuals all over the state of Alabama and to inspire underrepresented individuals to participate in the study through an educational and trust building process. Community partners (Supporting Information Table S1) foster this effort by introducing the CBR team to potential participants at different events throughout the state. Recruitment efforts included scheduling education sessions to cancer support groups, attending Relay For Life events in different Alabama counties, and participating in community partner BC‐specific events (i.e., walks and/or workshops). IRB‐approved flyers/brochures were disseminated at all recruiting events. These strategies ultimately identified individuals interested in study participation; subsequently, a CBR team member scheduled enrollment appointments for those who met the criteria and expressed interest in the study. Enrollment appointments were scheduled at the convenience of the study participant. In order to address transportation and other barriers limiting research participation, the CBR team traveled to the study participants for their enrollment appointments (Figure 1). Since April 2017, the Gene Machine has been used for CBR travel, which is a refurbished bus that serves as study advertisement and a mobile recruitment and enrollment station (Supporting Information Figure S1).

Upon study consent at a CBR enrollment session, similar to hospital recruitment, an individual shares demographic information, her/his personal and family history of cancer, and other cancer risk factors. From this information, a family pedigree is generated. Medical information about a participant's cancer diagnosis is also shared but, in this setting, medical reports are provided through the participant. CBR study participants also consent to a blood draw for DNA extraction (AU IRB #14‐335), which is carried out by a trained CBR team member. In circumstances when blood samples are not attainable/practical (i.e., individuals who had double mastectomies and lymph node removal), saliva samples can be provided. The CBR‐collected samples are assigned an alpha‐numeric code for laboratory use to protect participant confidentiality. Lastly, study participants agree to be contacted in the future for additional sampling and/or information pertaining to cancer risk, updates, and potential family member involvement. Upon enrollment, it is the job of the study proband to reach out to family members to inform of the study, gauge interest, and inquire about study involvement. Once interest is expressed and permission granted, the CBR team can contact family members for an enrollment appointment.

### 2.4 DNA bank and database

The Merner DNA bank and database protocol (AU IRB #14‐335) was established to organize the storage and use of collected information and samples. After samples are collected at enrollment sessions of protocols #14‐232 or #15‐111, they are transported to the AU laboratory for DNA extraction. Blood DNA is extracted following a protocol published by Miller, Dykes, & Polesky, (1988). The participant's DNA is then stored at 4°C in the DNA bank; the exact location of the participant's DNA is recorded in the database. The database only contains de‐identified information including the alpha‐numeric sample code along with demographic and medical information that corresponds to each sample/study participant. Furthermore, the database describes how each sample can be used in research.

## 3 RESULTS

**Figure 2:**
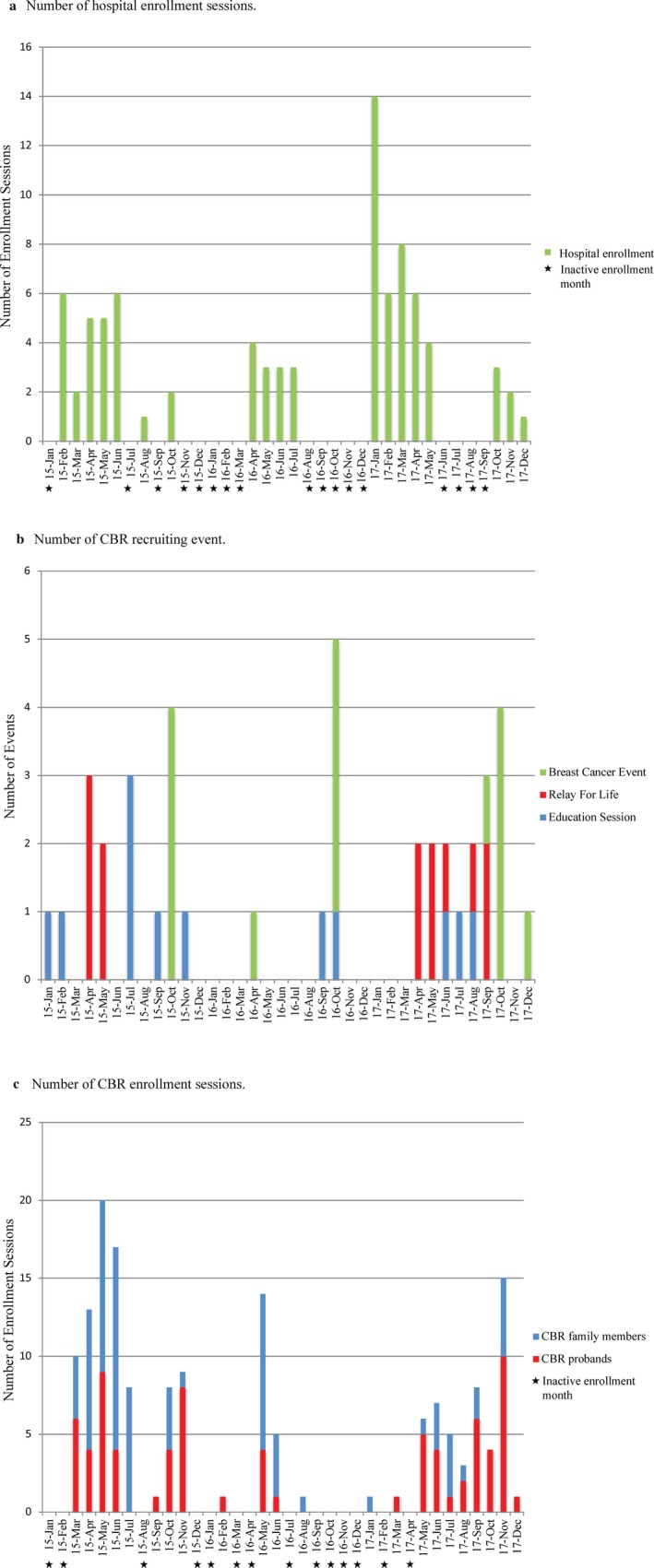
Recruiting events and enrollment sessions over the 36‐month period

Upon 36 months of recruitment and enrollment, the Alabama Hereditary Cancer Cohort has 242 individuals from 160 cancer‐affected families (Supporting Information Figure S2 and Figure 2). This includes 160 cancer probands and 82 cancer‐affected and unaffected family members from 27 different counties in Alabama (Supporting Information Figure S2 and Figures 1 and 2); 81% of the study participants’ counties of residence (22 of 27 counties) are completely medically underserved (Figure 1). Of all the cancer probands, 52% (*n* = 84) were recruited through hospital recruitment (Supporting Information Figure S2 and Figure 2a) and 48% (*n* = 76) through CBR (Supporting Information Figure S2 and Figure 2b,c). All family members were recruited through CBR (Supporting Information Figure S2 and Figure 2c). Overall, 62% (*n* = 99), 37% (*n* = 59), and 1% (*n* = 2) of the probands self‐reported being of European, African, and Asian descent, respectively. Ninety percent (*n* = 144) of the probands are BC cases (Supporting Information Figure S2A and Table 1).

**Table 1:** Summary of proband characteristics

Alabama hereditary cancer cohort		Breast cancer probands							Ovarian cancjpg" mimeTypeer probands					Prostate cancer probands				
		Total number of individuals			Individuals diagnosed ≤ 45 yoa	Individuals diagnosed > 45 yoa	Average age of BC onset^a^	Range of BC onset	Total	Individuals diagnosed ≤ 45 yoa	Individuals diagnosed > 45 yoa	Average age of OvC onset	Range of OvC onset	Total	Individuals diagnosed ≤ 45 yoa	Individuals diagnosed > 45 yoa	Average age of PC onset	Range of PC onset
		Female	Male	Total														
Hospital recruitment	African American	20	1	21	9	12	50.0	23–69	0	0	0	N/A	N/A	1	0	1	63	63
	European American	57	0	57	22	35	50.0	24–70	3	0	3	57	52–63	1	0	1	51	51
	Asian American	1	0	1	1	0	39.0	39	0	0	0	N/A	N/A	0	0	0	N/A	N/A
	Combined	78	1	79	32	47	49.8	23–70	3	0	3	57	52–63	2	0	2	57	51–63
CBR	African American	32	0	32	19	13	46.1	32–61	1	1	0	24	24	2	0	2	62.5	53–72
	European American	31	1	32	14	18	46.3	24–69	0	0	0	N/A	N/A	3	0	3	69.7	62–81
	Asian American	1	0	1	1	0	39	39	0	0	0	N/A	N/A	0	0	0	N/A	N/A
	Combined	64	1	65	34	31	46.1	24–70	1	1	0	24	24	5	0	5	66.8	53–81
Total	African American	52	1	53	28	25	47.6	23–69	1	1	0	24	24	3	0	3	62.7	53–72
	European American	88	1	89	36	53	48.7	24–70	3	0	3	57	52–63	4	0	4	65	51–81
	Asian American	2	0	2	2	0	39	39	0	0	0	N/A	N/A	0	0	0	N/A	N/A
	Combined	142	2	144	66	78	48.1	23–70	4	1	3	48.8	24–63	7	0	7	64	51–81

*Notes*

CBR: community‐based recruitment; yoa: years of age.

a If individuals were diagnosed with multiple primary BC tumors, the age of BC used in this table was their age at the first BC diagnosis.

### 3.1 Recruitment and enrollment

Although the recruitment efforts and enrollment sessions were carried out over a span of 36 months, all months did not receive equal efforts for recruiting and enrolling individuals into the study (Figure 2). Months in which resources (i.e., time and personnel) were allotted toward enrollment sessions are defined as active enrollment months (AEMs), whereas no efforts were made in inactive enrollment months. There were a total of 17 and 14 inactive enrollment months for hospital recruitment and CBR, respectively (Figure 2a,c).

### 3.2 Hospital recruitment

After the screening process, the research nurse contacted eligible individuals and approximately equal percentages accepted and declined participation. Accordingly, this 50% hospital participation rate resulted in the enrollment of 84 probands; 73% (*n* = 61), 26% (*n* = 22), and 1% (*n* = 1) are European, African, and Asian American, respectively (Supporting Information Figure S2A and Table 1). Of the 19 total AEMs, seven, four, and eight fell in 2015, 2016, and 2017, respectively, with an overall average enrollment of four individuals per AEM (Figure 2a). The majority of study participants enrolled in 2017 (*n* = 44; Figure 2a) with an average enrollment of six per AEM. The least successful enrollment year was 2016 with only 13 new study participants, averaging three per AEM (Figure 2a). Ninety‐four percent (*n* = 79) of the probands are BC cases with 49.8 years being the average age of onset (Table 1). Of the 79 BC probands, 72% (*n* = 57) are European American, 27% (*n* = 21) are African American, and 1% (*n* = 1) is Asian American (Supporting Information Figure S2A). One of the BC probands is an African American male. He was diagnosed at 42 years of age with moderately differentiated, infiltrating ductal carcinoma and has a family history of the disease. OvC and PC cases represent 4% (*n* = 3) and 2% (*n* = 2) of the probands, respectively (Supporting Information Figure S2A and Table 1).

### 3.3 CBR

Recruitment efforts involved presenting 12 education seminars to cancer support groups and attending 13 Relay for Life events as well as 15 other BC‐specific events (Figure 2b). The latter of which typically occurred in October, BC awareness month, and primarily involved attending the same community partner‐organized events each year. Most of the recruitment efforts occurred in 2015 and 2017 (Figure 2b). Overall, attending CBR recruiting events highly corresponded to AEMs (Figure 2b,c), except in October of 2016 when limited resources were allotted to study enrollment. In fact, overall, the least amount of recruitment and enrollment efforts were allotted for 2016 (Figure 2b,c). Of the 22 CBR AEMs, eight, four, and 10 fell in 2015, 2016, and 2017, respectively. With a total of 158 study participants who enrolled through CBR, the overall average enrollment rate was seven individuals per AEM (Figure 2c). The majority of the CBR study participants enrolled in 2015 (*n* = 86) averaging 11 per AEM. Both 2016 (*n* = 21) and 2017 (*n* = 51) averaged five new enrollees per AEM (Figure 2c).

A total of 76 CBR probands enrolled in the study of which 50% (*n* = 38), 49% (*n* = 37), and 1% (*n* = 1) are European, African, and Asian American, respectively (Supporting Information Figure S2A and Figure 2c). The majority of the probands enrolled in 2015 (*n* = 36) and 2017 (*n* = 34), averaging four and three new probands per AEM for each respective year. Only six probands enrolled in 2016 (Figure 2c). Overall, 20% were initially identified at an education session, 22% through a Relay for Life, and 22% at a BC event; the remaining 36% were informed of the study through word of mouth or general publicity (i.e., a newspaper article). However, this differed based on ethnicity. More African American probands enrolled in the study after attending an education session or meeting the CBR team at a Relay for Life event (30% and 27%, respectively) compared to European American probands (11% and 16%, respectively). Moreover, word of mouth/general publicity contributed to only 22% of the African American probands but 50% of the European American probands. Eighty‐six percent (*n* = 65) of the CBR probands are BC cases with 46.1 years being the average age of onset. Of the CBR BC probands, 49% (*n* = 32) are European American, 49% (*n* = 32) are African American, and 2% (*n* = 1) are Asian American (Supporting Information Figure S2A and Table 1). Through CBR, one European American male diagnosed with multifocal intraductal papilloma at 48 years of age enrolled in the study. In addition to BC‐, OvC‐, and PC‐ affected individuals (Supporting Information Figure S2A and Table 1), five unique cancer cases/probands enrolled through CBR when the individual had an apparent family history of BC, OvC, or PC (Supporting Information Figure S2A). This included three (one European American and two African American) females diagnosed with uterine cancer as well as one European American female diagnosed with colorectal cancer. The unique cancer cases also included an European American male who was diagnosed with squamous cell skin cancer at 65, melanoma at 70, and pancreatic cancer at 72 years of age (Supporting Information Figure S2A).

**Figure 3:**
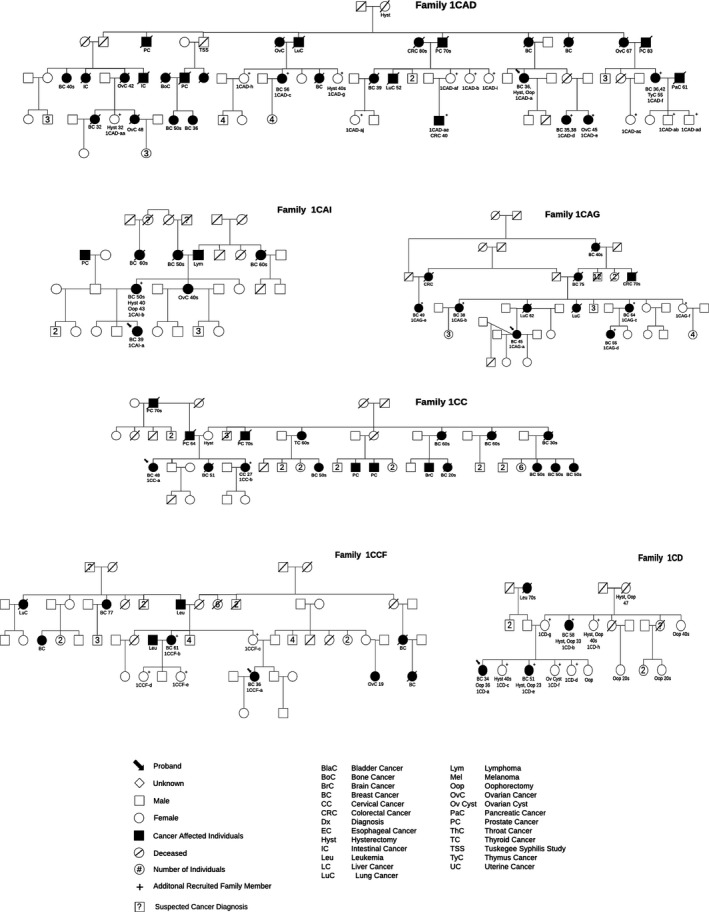
Selected African American pedigrees of families with multiple study participants

A total of 82 family members enrolled in the study through CBR (Supporting Information Figure S2), of which 50, 15, and 17 family members enrolled in 2015, 2016, and 2017, respectively (Figure 2c); family member enrollment rates were six, four, and two per AEM for each respective year. Despite that some family members were recruited along with their proband at the same recruiting event, family member recruitment was highly dependent on study participants reaching out and informing additional family members about the study. Of the 82 family members, 27 were cancer‐affected and 55 were unaffected individuals (Supporting Information Figure S2B). The majority (70%; *n* = 57) were African American, of which 95% (*n* = 54) were family members of BC probands. Overall, a total of 12 African American BC families with multiple cancer‐affected study participants have enrolled in the study (Supporting Information Figure S2B and Figure 3); the largest families are 1CAD and 1CAG ‐ each with six and five cancer‐affected study participants, respectively. Family 1CAD also has 10 cancer‐unaffected study participants, making it the largest enrolled family (Figure 3). European Americans represent 28% (*n* = 23) of the enrolled family members (Supporting Information Figure S2B). The majority (91%; *n* = 21) of the European American family members were of BC probands, composing a total of 15 families, four of which have multiple cancer‐affected individuals (Supporting Information Figure S2B).

## 4 DISCUSSION

With the extreme need to include underrepresented populations in medical research (Bustamante et al., 2011; Konkel, 2015; Spratt et al., 2016; Streicher et al., 2011), the establishment of the Alabama Hereditary Cancer Cohort is a timely and vital effort from which to gain insight. In order to focus on individuals with a predisposition to hereditary BC, recruitment criteria were established to identify affected individuals with hallmark characteristics such as a family history of BC and associated cancers and early ages of onset (Apostolou & Fostira, 2013; Chandler et al., 2016). A number of hereditary cancer syndromes exist for which BC is an associated cancer. Hereditary Breast and Ovarian Cancer (HBOC) Syndrome (Hall et al., 1990; Lynch & Krush, 1971; Lynch et al., 1972, 1974; Narod et al., 1991) is one such syndrome that is characterized by BC and/or OvC in multiple generations, as well as diagnoses under 45 years of age, women with multiple primary BCs or both BC and OvC, male BC, and/or a family history of certain other cancers, including PC, melanoma, and pancreatic cancer (Shiovitz & Korde, 2015). This recruitment effort targeted probands who were primarily diagnosed with BC, OvC, and PC, three associated cancers of HBOC. Furthermore, unique cancer cases with a family history of such cancers have been recruited. This strategy was developed in recognition that BC is typically not the only cancer noted on a hereditary BC pedigree. Thus, the recruitment criteria allow for the inclusion of individuals/families who may have a genetic predisposition but could have been excluded from a study solely enrolling BC probands. For instance, it allows alternate cancer probands to enroll into the study who are from families that have experienced BC mortalities or have family members diagnosed with BC but unwilling to participate. It also recognizes families with a higher proportion of males to females that are more likely to observe PC over BC. Ultimately, with the main goal of identifying BC genetic risk factors, BC cases represented the majority (90%) of the probands recruited into the study. Nonetheless, in order to expand OvC and PC proband recruitment, additional effort needs to be made, such as committing more time identifying individuals diagnosed with such cancer types in both the hospital and community settings. Regarding the latter, partnering with OvC and PC support groups will be key since, to date, our community partners are primarily BC support groups. Additionally, it is important to recognize other hereditary cancer syndromes, including Cowden, Li‐Fraumeni, Hereditary Diffuse Gastric Cancer, and Peutz–Jeghers Syndromes that are all associated with different types of inherited cancers in addition to BC (Shiovitz & Korde, 2015). Overall, the particular gene/mutation involved in pathogenesis dictates the predisposition to particular cancer types and the cancer patterns observed in a family. Ultimately, in order to encompass all possible inherited BC syndromes and inheritance patterns, it is important to keep the definition of family history broad, asking study participants to acknowledge all cancers that they are aware of in their family, and recognize and enroll unique cancer probands who have a family history of BC.

In order to offer BC genetic research participation to individuals in the medically underserved state of Alabama who would not normally be given the opportunity to participate in such a research study, both hospital recruitment and CBR mechanisms were established. Both recruitment mechanisms were instrumental in enrolling individuals into the study. Together, they led to the enrollment of 242 study participants who provided information and samples that have been incorporated into the Merner DNA bank and database. This includes 160 cancer probands (90% of which were BC probands) and 82 family members. Hospital recruitment is the most traditional mode of recruitment for a genetic research study (Salowe et al., 2017). Our seminal hospital recruitment efforts involved identifying and enrolling patients at EAMC, a regional hospital in Lee County that serves 6 medically underserved Alabama counties. Despite that intermittent enrollment periods due to unforeseen circumstances at EAMC resulted in inactive enrollment months, one research nurse devoted approximately 0.25 full‐time equivalent (FTE) toward this project during AEMs. Thus, during times of active enrollment, the typical 10 hours of weekly effort toward the project was divided into approximately seven hours of eligibility screening and contacting patients, and approximately three hours of enrollment appointments and paper work/data entry. Overall, a much lower participation rate (~50%) was observed compared to reports from other hospitals that have enrolled in genetic studies (with claims as high as 100%); however, it is important to note that participation rates are known to vary between hospitals, and when Helgesson, (2011) compared factors that could influence such participation rate differences, the actual site of recruitment was determined to be the most important factor. Despite that the site‐specific study coordinator's motivation, demeanor, knowledge, and ability to communicate and build trust can influence such rates (Helgesson, 2011), it is important to recognize that the EAMC service area is medically underserved and individuals in the area have very rarely been offered to participate in a research study. Plus, many individuals have been negatively and justifiably influenced by historical events that cast doubt on even the most well‐intended efforts (Brandt, 1978), which is likely another contributing factor. An investigation into the exact factors that influenced the initial EAMC participation rate and ways to improve is pertinent. Overall, upon being offered study participation, 84 probands enrolled into the study at EAMC during 19 AEMs. Therefore, an overall average of four EAMC study participants enrolled per AEM, which ranged from an average of three to six individuals per AEM for each year of the study. Stemming back to the potential impacts of a study coordinator, the different yearly averages ultimately correlated with the assigned research nurse. On another note, the percentages of European American (73%), African American (26%), and Asian American (1%) probands that enrolled into the study at EAMC closely represented the racial demographics of the cancer center's patient population, being 65% European American, 33% African American, and 2% other (averaged over the three years of the study). Interestingly, this is contrary to other clinic‐based studies that typically have a difficult time enrolling ethnic minorities (Helgesson, 2011; Salowe et al., 2017).

CBR has been suggested to be an effective method to recruit medically underserved and underrepresented racial/ethnic minorities into research studies (Greiner et al., 2014); thus, we designed a CBR mechanism to overcome barriers known to hinder research participation (Brown, 2004; Farmer et al., 2007). In order to reach out to individuals all over the state, an educational and trust‐building recruitment process was established that involved traveling to different Alabama counties/communities. Specifically, four unique modes of recruitment were developed: offering education seminars to cancer support groups, attending Relay for Life events, participating in BC‐specific events, and word‐of‐mouth/general publicity. Presenting education seminars and attending both Relay for Life and BC‐specific events were all essential to the success of this project, since each mode yielded similar enrollment of CBR probands. Although word‐of‐mouth/general publicity led to the enrollment of the largest portion of the CBR probands (36%) compared to the other three modes of recruitment individually, this mode led to the enrollment of a smaller portion of African American probands (22%) compared to European American probands (50%). The CBR team, which is mainly composed of European Americans, likely influenced the discrepancy in ethnicities recruited through word‐of‐mouth/general publicity since the ethnicity of the recruitment team has been reported to greatly influence participation of underrepresented individuals in medical research studies (Farmer et al., 2007). Thus, diversifying the CBR team to adequately represent the targeted population will likely help. However, interestingly, CBR ultimately enrolled equal numbers of European and African American BC probands; thus, the trust building and educational components of the other modes of recruitment highly influenced African American enrollment. In fact, most African American probands were recruited after attending an education session or meeting a CBR team member at their local Relay for Life. This was accomplished by targeting African American BC support groups for education sessions and choosing to attend Relay for life events in predominantly African American communities; hence, why the proband ethnic proportions do not match the state racial demographic (United States Census Bureau: Alabama Quick Facts, 2017).

The initial CBR efforts were carried out on an extremely small‐scale. In 2015, the principal investigator (PI) and a graduate student carried out CBR. Our first community partner, SISTAs CanSurvive Coalition, fostered the invitations to our first education seminars. Furthermore, attending Relay for Life events not only identified some of our initial CBR study participants but also facilitated additional partnerships, which subsequently resulted in more invitations to education sessions and BC‐specific events. In 2015, despite not measuring the exact participation rate, the recruitment and subsequent enrollment of 86 study participants through eight AEMs (averaging 11 individuals per AEM), as well as all other related tasks (such as relationship building, modifying protocols/dissemination materials, traveling, DNA extractions, database management, etc.), consumed the majority of the PI's (70%) and graduate student's (30%) workload. However, the efforts put forth in that inaugural year were necessary to demonstrate proof of concept. In 2016, with the newly established cohort, the PI's focus changed to seeking research funds and initiating genetic analyses hence the observance of so many inactive enrollment months. In 2017, funds were obtained to hire a recruitment coordinator who worked 0.67 FTE and allotted approximately 20% effort each toward recruiting, enrolling, traveling (on the Gene Machine), extracting DNA, and managing the DNA bank and database. Upon training, the recruitment coordinator independently enrolled individuals into the study from May to December (averaging six study participants per AEM through that period). Ultimately, over a total of 22 CBR AEMs, 158 individuals enrolled in the study for an overall average enrollment of seven individuals per AEM. Moving forward, the goal is to expand the CBR team and designate duties to make CBR as efficient as possible.

Similar to the initial efforts of the Carolina Breast Cancer Study (Newman et al., 1995), our CBR efforts involve traveling to individuals for enrollment appointments. We travel all over the state to enroll eligible individuals in order to overcome logistic barriers to research participation; currently, we have enrolled individuals from 27 counties. In addition to proband enrollment, this component of CBR has also proven to be an excellent approach to enroll large families for genetic analyses. Similar to the approach used by the Family Information Service described by Dr. Henry Lynch in 2001 (Lynch, 2001), the CBR team coordinates and attends large family gatherings to recruit and enroll a large number of family members in a single session. For example, the CBR team receives invitations to family reunions, which are phenomenal events to provide an education seminar and enroll individuals as family members reunite. However, if a single session is not ideal for family members due to barriers in transportation and/or conflicting time‐commitments, the CBR team also travels to different towns to recruit individuals from the same family. For instance, the team traveled to towns in three different counties to recruit members of family 1CAG in Figure 3. Overall, African Americans represented the majority (70%) of family members that enrolled in the study; thus, again, reiterating that CBR is a great mechanism to involve African Americans in genetic research and provides a collection of unique families/individuals for analyses. Furthermore, since most of Alabama is rural, travel includes visiting isolated communities that are likely enriched for ancestral genetic mutations. Thus, by traveling to these communities for recruitment, the CBR team can cater to underserved populations as well as harness their genetic potential and detect ancestral mutations in seemingly unrelated probands/families. After all, studying cohorts derived from isolated populations is currently an extremely palatable approach toward BC susceptibility gene discovery (Chandler et al., 2016).

The recruitment mechanisms and stemming biobank also allows the investigation of particular cancer disparities. Firstly, African American BC genetics is vastly understudied and less understood compared to European American BC genetics (Churpek et al., 2015). Studying African American hereditary BC is a priority of this study since African American women are more often diagnosed with an aggressive and less treatable BC sub‐type and have a higher incidence rate of BC under the age of 40 compared to European Americans (ACS, 2014). Similarly, African American males are more susceptible to PC compared to European Americans, and normally diagnosed at a younger age and with larger tumors (Zenka, 2012). Thus, considering that (1) an early age of onset is a hallmark of hereditary cancer, (2) hereditary BC is associated with an increased risk of PC (Berliner & Fay, 2007), and (3) the Black Women's Health Study has demonstrated there is a strong familial component of African American BC (Palmer, Boggs, Adams‐Campbell, & Rosenberg, 2009), it is likely that genetic risk factors contribute toward the higher incidence rate of early onset and aggressive BC and PC in African Americans and that the two disparities are genetically‐linked. This potential link will be investigated, especially with the success of CBR regarding African American enrollment. Additionally, the families that have been recruited, such as our largest African American family, 1CAD, are excellent examples of both BC and PC segregating in the same family, highlighting our resources and the practicality to investigate the genetic overlap.

## 5 CONCLUSIONS

This initial report details the protocols that were established and carried out to enroll underrepresented individuals into a hereditary BC cancer genetic study and the subsequent development of a biobank from which samples can be used in future independent and collaborative cancer genetic studies. It specifically highlights the rudimentary accomplishments made during the first three years of the project and provides insight on how to continue and expand the efforts. A hospital recruitment protocol was established for its efficiency. It is the most standard mode of recruitment due to the ease of identifying study participants, obtaining complete medical records, and carrying out enrollment appointments. Therefore, in order to expand this efficient mode of recruitment, the protocol was strategically designed to add collaborating hospitals through IRB reliance agreements. However, it is important to note that site‐specific enrollment rates will vary greatly depending on the percent FTE allocated to the project as well as each study coordinator's personality. Furthermore, due to Alabama being a significantly medically underserved state with double the national percentage of self‐identifying African Americans, it was crucial to adapt and develop an alterative recruitment method. CBR focused on overcoming recruitment barriers, enabling our team to connect with even more underrepresented individuals in the state. We aspire to grow similarly to the Carolina Breast Cancer Study (Newman et al., 1995), which has continued to function for over 20 years and now has a large staff of interviewers, nurses, and technicians committed to the project. Currently, we plan to continue to work closely with our partners and stay connected with the community as we travel to events, education seminars, and enrollment appointments on the Gene Machine, which now has a strong presence on social media and has begun to unofficially brand our CBR efforts providing a new marketing component and mode of recruitment. Overall, we have learned that the effort required to include underrepresented individuals in research is immense and challenging. It is a vital effort that should no longer be underappreciated.

## CONFLICT OF INTEREST

The authors disclose no potential conflicts of interest.

## AUTHOR CONTRIBUTION

Conception and design: N.D.M.; Development of methodology: M.R.B., B.J., and N.D.M.; Writing, review and/or revision of manuscript: M.R.B., A.S., A.L.W.H., M.S., and N.D.M.; Hospital‐based recruitment: A.D., H.D., B.J., E.S., and N.D.M.; Community‐based recruitment outreach/education: M.R.B., E.S., A.S., M.S., E.P.B., E.J., K.D., A.L.W.H., S.M.O., S.S., K.S., S.B., and N.D.M.; Community‐based recruitment enrollment: M.R.B., E.S., E.P.B., E.J., and N.D.M.; DNA extractions: M.R.B., E.S., E.J., and E.P.B.; Database management: M.R.B., E.S., and N.D.M.; Mutation validation: M.R.B. and N.D.M.
