# Supplementary material for: Establishment of the Alabama Hereditary Cancer Cohort ‐ strategies for the inclusion of underrepresented populations in cancer genetics research
Source: Mol Genet Genomic Med. 2018 Jul 1;6(5):766–78. doi: 10.1002/mgg3.443 (PMC6160710; doi:10.1002/mgg3.443)
Supplement: Supplementary file 1 [file MGG3-6-766-s001.docx]

**Supporting Information**

***Title***: Establishment of the Alabama Hereditary Cancer Cohort - strategies for the inclusion of underrepresented populations in cancer genetics research.

***Authors:*** Bishop MR, Shah A, Shively M, Huskey ALW, Omeler SM, Bilgili EP, Jackson E, Daniell K, Stallworth E, Spina S, Shepp K, Bergstresser S, Davis A, Dean H, Gibson J, Johnson B, Merner ND

**Supporting Information Table S1:** Descriptions of five IRB-approved community partners.

| **Name** | **Mission** | **Research Involvement** |
| --- | --- | --- |
| Alabama Breast and Cervical Cancer Early Detection Program (ABCCEDP) | An Alabama Department of Public Health (ADPH) Initiative that provides free BC and cervical cancer screenings for underserved women who meet certain poverty eligibility guidelines. | Disseminates an IRB-approved information letter about the research study to women who were diagnosed with BC through the ABCCEDP. |
| Macon M.E.A.N.S for Cancer - Support Group | An African American BC support group in Tuskegee, Alabama that focuses on mentorship, education, advocacy, and nutrition support. | Disseminates brochures and flyers about the research study and invites the CBR team to support group meetings. |
| SISTAs Can Survive Coalition (SCSC) | An African American BC support group in Montgomery, Alabama that provides a culturally sensitive focus to combat and cope with cancer. Their mission is to increase cancer survivorship by improving quality of life of survivors amongst the medically underserved. | Disseminates brochures and flyers about the research study and invites the CBR team to support group meetings and speak at their annual BC walk. Founder, Carrie Nelson, is a patient advocate on this grant proposal and offers a patient perspective on the study design. |
| Working to Help Those In Pink (W.H.I.P.) | An African American BC support group in east Alabama that offers help to survivors, family members, and supporters of BC. | Disseminates brochures and flyers about the research study, and invites the CBR team to support group meetings and an education booth at their annual BC walk. |
| Young Breast Cancer Survivorship Network (YBCSN) | An initiative out of the University of Alabama at Birmingham School of Nursing that provides targeted online resources to young women facing BC, with a central focus to improve their quality of life through education, support, and networking. | Disseminates brochures and flyers about the research study, and has invited the CBR team to support group meetings and their annual BC workshop. |

**Supporting Information Figure S1:** The Gene Machine, a mobile recruitment and enrollment station.


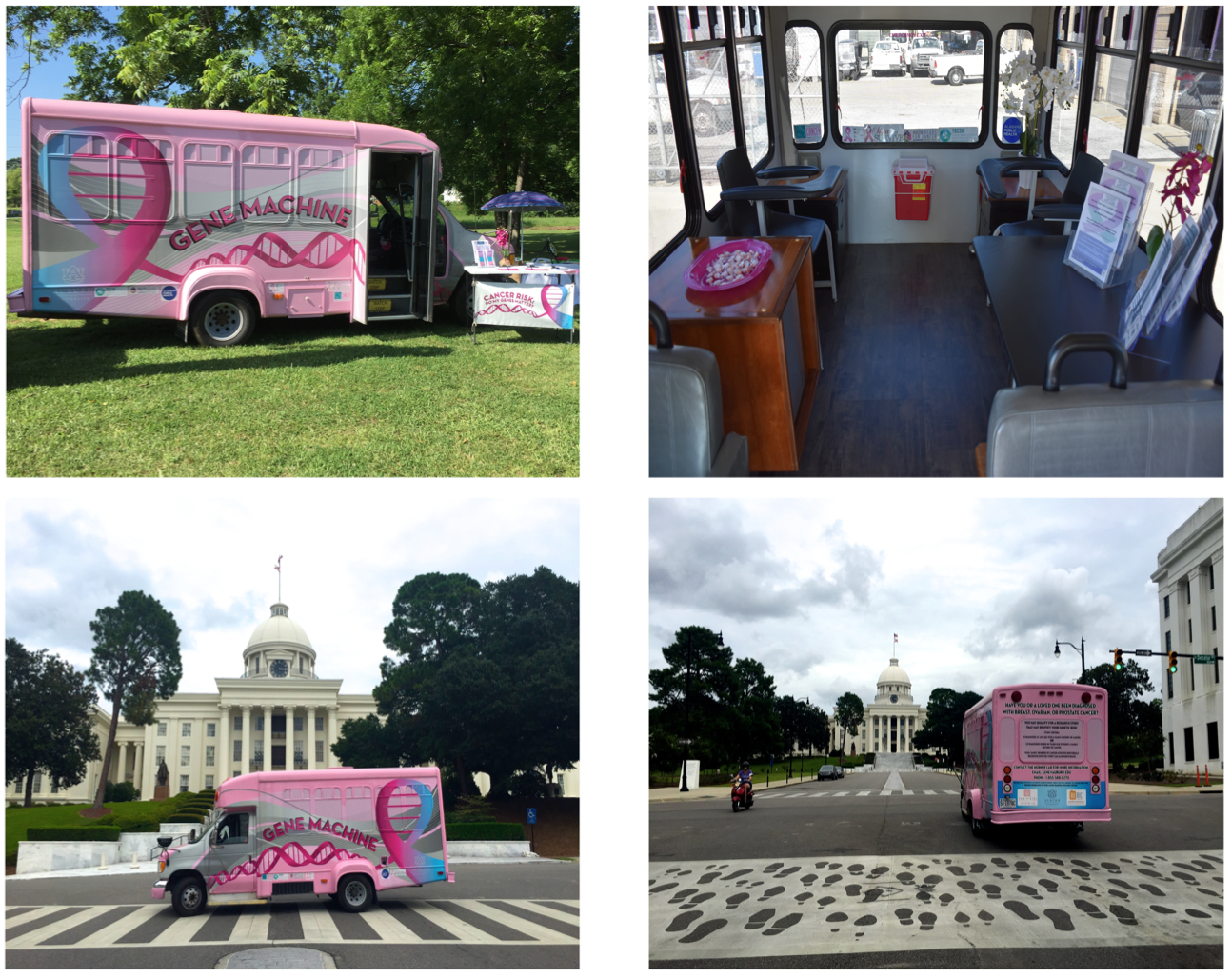

 **Supporting Information Figure S2:** Flowchart of samples in the Merner DNA Bank. Panel A illustrates how the DNA samples in the bank are divided into recruitment mechanism, participation category (proband or family member), ethnicity, and cancer type. Panel B further explains characteristics of additional family member who have been recruited into the study.
