# Supplementary figures and images for: Establishment of the Alabama Hereditary Cancer Cohort ‐ strategies for the inclusion of underrepresented populations in cancer genetics research
Source: Mol Genet Genomic Med. 2018 Jul 1;6(5):766–78. doi: 10.1002/mgg3.443 (PMC6160710; doi:10.1002/mgg3.443)

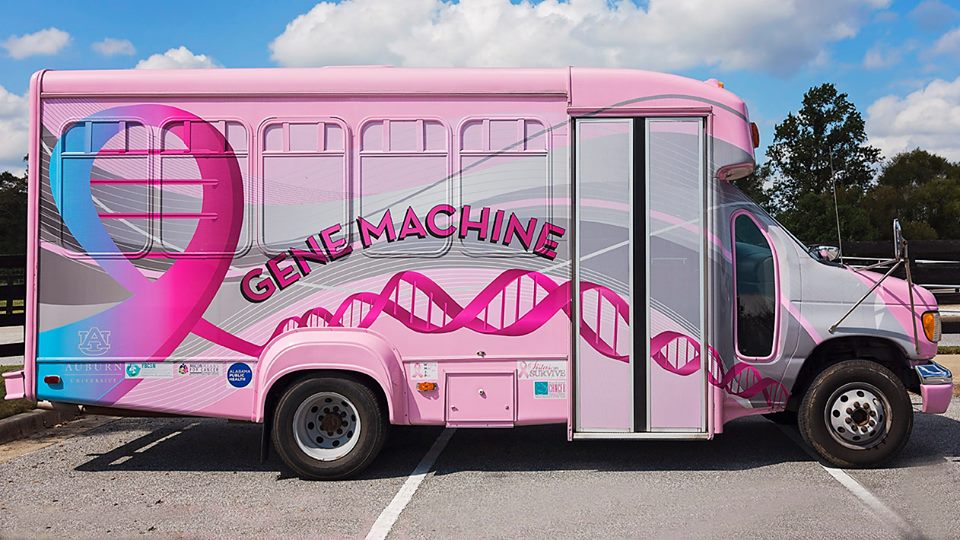

Supplement: Supplementary file 2 [file MGG3-6-766-s002.jpg]
